# Beyond bridging the know-do gap: a qualitative study of systemic interaction to foster knowledge exchange in the public health sector in The Netherlands

**DOI:** 10.1186/s12889-015-2271-7

**Published:** 2015-09-19

**Authors:** Francine van den Driessen Mareeuw, Lenneke Vaandrager, Laurens Klerkx, Jenneken Naaldenberg, Maria Koelen

**Affiliations:** Radboud University Medical Center, Department of Primary and Community Care, Geert Grooteplein 21, 6525 EZ Nijmegen, The Netherlands; Health and Society Group, Department of Social Sciences, Wageningen University, Hollandseweg 1, 6706 KN Wageningen, The Netherlands; Knowledge, Technology and Innovation Group, Department of Social Sciences, Wageningen University, Hollandseweg 1, 6706 KN Wageningen, The Netherlands

**Keywords:** Public health, Health promotion, Innovation system, Know-do gap, Implementation, Knowledge exchange, Stakeholders

## Abstract

**Background:**

Despite considerable attention currently being given to facilitating the use of research results in public health practice, several concerns remain, resulting in the so-called know-do gap. This article aims to identify the key tensions causing the know-do gap from a broad perspective by using a systemic approach and considering the public health sector as an innovation system.

**Methods:**

An exploratory qualitative design including in-depth semi-structured interviews was used, with 33 interviewees from different actor categories in the Dutch public health innovation system. The analyses employed an innovation system matrix to highlight the principal tensions causing the know-do gap.

**Results:**

Seven key tensions were identified, including: research priorities determined by powerful players; no consensus about criteria for knowledge quality; different perceptions about the knowledge broker role; competition engendering fragmentation; thematic funding engendering fragmentation; predominance of passive knowledge sharing; and lack of capacity among users to use and influence research.

**Conclusions:**

The identified tensions indicate that bridging the know-do gap requires much more than linking research to practice or translating knowledge. An innovation system perspective is crucial in providing information on the total picture of knowledge exchange within the Dutch public health sector. Such a system includes broader stakeholder involvement as well as the creation of social, economic, and contextual conditions (achieving shared visions, building networks, institutional change, removing financial and infrastructural barriers), as these create conducive factors at several system levels and induce knowledge co-creation and innovation.

## Background

Public health research has the potential and goal to improve people’s health and wellbeing. Internationally, organisations funding public health research are concerned that research-based knowledge about public health issues is not sufficiently used to enhance (public) health policy and practice (e.g., to inform evidence-based interventions) [1, 2]. This means that their investments in research are not reflected in societal benefits [3]. The lack of sufficient knowledge exchange between research, policy, and practice is considered to be a major underlying cause of this problem [4–7] and is often referred to as the know-do gap [8]. Improving knowledge exchange is therefore a priority area for public health research funding agencies, and several studies have examined this topic [2, 9–11].

Knowledge exchange processes in the public health context to stimulate change and innovation (e.g., in interventions, protocols) are increasingly considered to be dependent on the broader, multi-actor context in which these processes take place [12–16]; this implies that they should be seen as interactive and systemic processes rather than linear processes in which research findings are ‘pushed’ towards, and ‘translated’ for, the intended user by knowledge brokers or translators [10, 11]. A new public health approach, for example a healthy communities approach, requires changes in the way neighbourhoods are planned and built, coordination between local health and welfare professionals, intersectoral governance, and so forth [17], and involves different types of knowledge (such as research-, practice-, or experience-based knowledge) [18]. On the assumption that such change and innovation processes are co-evolutionary processes in which multiple actions need to happen simultaneously to effectuate change [16, 19], the embedding of new knowledge often implies broader adaptations in for example work procedures, incentive structures, and even physical infrastructure (i.e., medical equipment) [12, 20]. A systemic and co-evolutionary view on innovation also implies that a change in one part of a system (or sector) may have an impact - sometimes unintended - on another part of the system [15, 21]. Such views on innovation as a co-evolutionary process that depends on coordinated systemic interactions among networks of multiple actors are now recognised in the innovation systems literature [12–14, 16]. This holistic perspective on multi-actor collaboration for innovation recognises the key importance of the exchange of different types of knowledge of different actors in systems, related in this context to the multiple changes in public health systems that are needed for innovation.

An innovation system may be national, regional, or sectorial [22, 23] and has been defined as: “a network of organisations, enterprises, and individuals focused on bringing new products, new processes, and new forms of organisation into economic use, together with the institutions and policies that affect the way different agents interact, share, access, exchange, and use knowledge” [24] (pp. 6–7). Within this study, the public health sector and its knowledge exchange processes are seen as an innovation system in which innovations are generated and implemented, i.e., diffused and disseminated, through the interaction of multiple stakeholders. Although bringing new products, processes, and forms of organisation into economic use may not be the foremost goal of public health, as it mostly caters for public goods such as disease prevention and health promotion, it has been recognised that public health systems can be seen as sectorial innovation systems [21, 25, 26]. However, the perspective of innovation systems has not yet been used as an analytical tool for investigating the know-do gap.

### Aims and analytical approach

On the assumption that the public health sector is an innovation system, the aim of this study was to apply an analytical framework based on innovation systems thinking to unravel the key tensions and underlying mechanisms causing the know-do gap within the Dutch public health sector in order to improve knowledge exchange. Existing literature in the field of mobility, agriculture, and water management offers matrices that can be used to map main characteristics of innovation systems [27–30]. Klein Woolthuis et al. developed a matrix that distinguishes actors and conditions within an innovation system that influence actor collaboration for innovation [28]. We now explain the analytical framework inspired by innovation systems thinking, focusing on the actors involved and conditions for their interaction.

As regards their contribution to the knowledge generation and exchange that underlies innovation, actors in innovation systems can be categorised on the basis of the role they play within the system: knowledge users, knowledge producers, intermediaries, and actors responsible for preconditions. Knowledge producers are actors who literally produce research-based, practice-based, or experience-based knowledge that can be shared with others. Different types of knowledge may be produced by different kinds of actors. Accordingly, knowledge users literally apply (use) research-based, practice-based, or experience-based knowledge to improve their practice [31]. The literature on knowledge exchange traditionally distinguishes knowledge producers and knowledge users, whereas the literature on innovation systems, including knowledge exchange processes, also takes into account intermediaries and actors responsible for preconditions [19, 22, 23, 32]. Intermediaries link different kinds of knowledge and/or different actors (e.g., brokering knowledge and/or linking users with producers), and those actors are often referred to as knowledge brokers [33, 34]. Actors responsible for preconditions facilitate knowledge exchange (e.g., by providing resources, competencies) and/or direct what knowledge is being produced [3, 19, 22, 23, 32]. Practical examples of actors responsible for preconditions include research funders and science and public health policymakers.

According to Klein Woolthuis et al. [28], the conditions that influence actor collaboration for innovation are categorised as infrastructural, institutional, interactional, and capability conditions. To assess innovation system performance, these conditions can be studied in a structured way to identify innovation system merits (positive) or innovation system failures (negative). In the public health context, these conditions would imply the following.

#### Infrastructural conditions

Concern physical infrastructure such as geographical distances between research institutes – often acting nationally – and local health professionals [5, 18] and knowledge infrastructure comprising science, knowledge brokering or consultancy, education, and attribution of roles.

#### Institutional conditions

Can be split into hard and soft institutions. Hard institutions refer to formal mechanisms that hinder or stimulate innovation, such as regulatory frameworks and funding schemes for obtaining funding for health promotion activities or research [5, 18]. Soft institutions concern norms and values. These relate to perceptions of the way business should be done, ideas on what is good knowledge, and incentive and reward systems. Green et al. exemplify this by showing that scientists are often more oriented towards international audiences than towards the needs of health professionals or the local public [18].

#### Interactional conditions

Address networks that are either (too) strong or (too) weak. Strong networks entail intensive cooperation, which can be very productive in terms of complementary knowledge, and so forth. In The Netherlands for example, several academic collaborative centres have been set up; these are structural collaborations between local governments, universities, and municipal health services [5, 35]. On the other hand, actor groups that cooperate very intensively may become inward looking and lack contacts outside their network that may have the potential to provide new insights. Such strong networks typically lack bridge builders to connect them to other networks. Weak networks reflect the opposite, in that new contacts may lead to new insights that stimulate innovation (for example single occasions of scientific advisory work in policy contexts) [35]. The risk with weak networks is that they may never mature to a point at which parties understand one another and build the trust required to cooperate successfully.

#### Capability conditions

Concern entrepreneurship and adequate staff qualifications. Organisations have to possess the necessary skills and resources to internalise new knowledge and technologies, and assess their value and applicability to the organisation [28]. An example of increasing capabilities is when researchers improve their competence to broaden the societal relevance of their research [5].

**Fig. 1:**
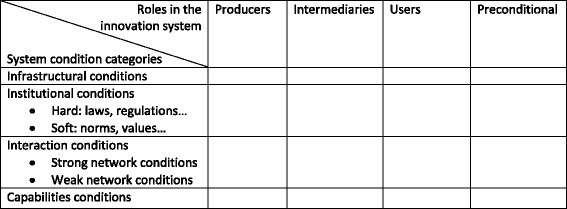
The innovation system matrix (after: Klein Woolthuis et al [28]). The first row presents the different actor roles in the system (producers, intermediary, users, preconditional). The first column presents the system condition categories in which identified tensions can be categorised

Klein Woolthuis et al’s matrix [28], combining the conditions and actor roles existing in an innovation system, is shown in Fig. 1.

This study uses this matrix in order to answer the following questions:

*What are the key tensions and underlying mechanisms explaining the know-do gap in the Dutch public health sector?*

*How do these tensions relate to system conditions of an infrastructural, interactional, institutional, and capabilities nature and actor roles?*

## Methods

### Design

This study follows an exploratory qualitative design. Individual in-depth semi-structured interviews were chosen as data-collection method because of the anticipated delicacy of the information sought from the interviewees, and methods sensitive to personal experiences were required.

### Selection of interviewees

Actors (organisations) that play a significant role in the public health innovation system were purposively sampled using stakeholder analysis, taking into account the background of the actor (research/policy/practice and/or public/private/NGO) and the actor level (national/regional/local). This was done in order to capture views and experiences from every part of the public health innovation system. First, a list of 60 stakeholders was drawn up, containing stakeholders from all backgrounds and levels. These stakeholders were asked to reply to an e-mail asking them about the knowledge they used (where did it come from?) and their job description. The 23 replies to these e-mails provided us with information about the most consulted sources for knowledge and the backgrounds and levels of actors. From this information, 35 relevant actors were identified. From these, representatives with decision-making authority were selected. A total of 33 interviewees representing 34 organisations (one interviewee worked for two relevant organisations) were included.

Table 1 provides an overview of the organisations represented.

**Table 1:** List of organisations that provided participants for the study. Thirty-three organisations (actors) participated in the study. One representative was interviewed from each of the 34 organisations presented in the table (one interviewee represented two organisations)

- → 3 universities (departments: health and nutrition, health and society, healthcare innovation)
- → 2 universities of applied sciences (departments of: healthcare innovation; health, behaviour, and society)
- → 6 research and training institutes (providing knowledge and sometimes training suitable for municipalities, municipal health services, schools, health professionals) on:
• → health promotion and public health
• → youth care
• → sports and physical activity
• → nutrition and food safety
• → mental and addiction care
• → alcohol policy
- → Academic collaborative (collaboration between municipal health service and university to jointly create knowledge on health promotion)
- → Public–private collaborative on overweight, joining together governmental organisations, private organisations (e.g., food industry), knowledge institutes
- → Municipality
- → 3 municipal health services (1 in an urban region, 2 in more rural areas)
- → Regional provider of mental healthcare
- → Supermarket headquarters (marketing department)
- → Communication/consultancy agency on food agriculture and health
- → Consultancy agency on innovation processes within public health/healthcare
- → 2 health insurance companies
- → Ministry of Health, Welfare, and Sport
- → Governmental institute promoting effective health promotion
- → Governmental institute supporting health policy by formulating public health prospects
- → Governmental health research funding agency
- → Healthcare inspectorate
- → Health council
- → 5 professional/umbrella associations of:
• → municipalities
• → care providers
• → municipal health services
• → general practitioners
• → dieticians

### Procedure

Thirty-five representatives of relevant actors were contacted by telephone to invite them for an interview and received information about this study by e-mail. Two representatives were not able to participate for practical reasons (lack of time, job switch).

The interviews were conducted between March and June 2009, by one of the authors (FDM). The interviews took place at a location chosen by the interviewee: interviewee’s workplace or in a public place such as a café. The average interview length was 90 min.

With the interviewees’ permission, all interviews were recorded (using a digital tape recorder) and transcribed afterwards. Interviewees received full transcripts of their interview and were asked to approve them, after which transcripts were anonymised. None of the interviewees asked for major revisions to their transcript.

### Interview topics

The interview topics included factors that participants perceived as facilitating or hindering with regard to knowledge exchange within, and the functioning of, the public health innovation system in The Netherlands. The interviews focused broadly on how the different actors in the system identified problems, what type of knowledge they used, produced, and exchanged, and how and why this was done. The topic list also included contextual conditions and the capacity of interviewees and their respective organisations to influence research agendas and make use of available knowledge. Table 2 gives an overview of the interview topics, accompanied with some examples of interview questions.

**Table 2:** Interview topics and related interview questions. The left column gives an overview of the interview topics. The right column shows the questions asked during the interviews relating to the topics

Interview topics	Questions posed to the interviewees (for each topic)
Occupation of interviewee and general information	- → Please describe your current occupation
	- → Please describe your organisation:
	• → Which role(s) does your organisation play within the Dutch public health sector?
	• → What does your organisation want to achieve and why?
Searching for information	- → In what situations do you need information?
	- → Do you consider searching for information to be one of your key tasks?
	• → How do you think others perceive your tasks?
	- → If you search for information, what sources do you use?
	- → What kind of information are you mostly searching for (thematic, methodological, other)?
	- → What criteria do you use for assessing the information you need? (What makes information useful for you/your organisation?)
	- → Are there other organisations that make information ready for use?
	• → What kind of organisations and what exactly do they do?
	- → What do you think of the available information? (quality, access,…)
	• → To what extent do you/does your organisation have influence on the availability and type of available information? Why?
	- → How do you think the process of searching for information could be made easier?
Processing information	- → Generally, for what do you use the information obtained? Why?
	- → How important is the information for you/your organisation? What factors influence this?
	- → Do you (or others) need to adapt the obtained information before you can use it?
	- → Who else uses this information (within and outside your organisation)?
Producing information	- → In what situation are you/is your organisation involved in producing information? Please describe the process:
	• → By whom (if applicable) are you involved?
	• → For what reason and in what stage of information production are you involved?
	• → What kind of information?
	- → What do you consider to be your responsibilities regarding producing information?
	• → Is it one of your key tasks?
	• → How do you think others perceive your role in producing information?
	- → Do you/does your organisation also collect or produce information for others? Why? What kind of information? Please describe such a situation:
	- → Do other organisations expect you to produce information? Why? What organisations?
	- → If you produce information for others, for whom and how are the others involved?
Sharing information	- → If you produce information for others, how is it used by others, and by whom?
	• → What is your influence on the use of information by others? Why? Would you like to have more influence?
	- → Do you ever transfer information produced by others, to others? Please describe such a situation:
	• → What exactly did you do? What steps did you take?
	• → What factors facilitated this information-sharing process?
	- → Do you think others expect you to share information?
Knowledge exchange in general	- → Generally speaking, how would you describe knowledge exchange within the Dutch public health sector?
	• → What factors influence it and how?
	- → What should be done by whom to improve knowledge exchange?
	• → What is needed? (structures, competencies …)?
	• → What role could you play in this?
	- → What do you consider to be knowledge?
	- → What else would you like to mention regarding knowledge exchange within the Dutch public health sector?

### Analytical process

The analytical process was supported by the use of software package ATLAS ti. for qualitative analyses (scientific software development).

**Fig. 2:**
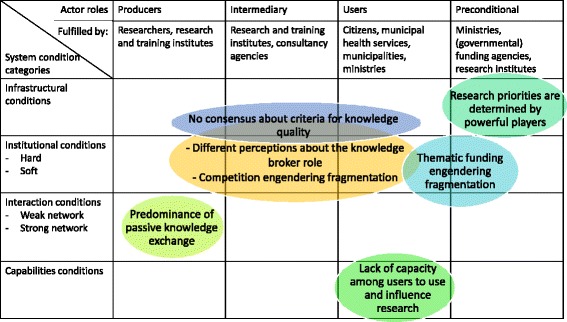
The innovation system matrix for the Dutch public health sector = area in which tension occurs.  The first row shows which actors - according to our findings - fulfil which actor roles. The location within the matrix of the identified tensions is defined by the system condition category to which the tension belongs and the domain of actor roles that cause and/or potentially resolve the tension (not necessarily reflecting the opinions of the actors involved or affected)

The data were analysed in two steps. First, the data were coded using a coding scheme that included codes relating to knowledge exchange, referring to the type of knowledge (research-based, practice-based, experience-based, or other) that was exchanged (‘what’), the actors exchanging knowledge (‘who’), in what way knowledge was exchanged (‘how’), and for what reasons knowledge was (not) exchanged (‘why’). This preparatory step was carried out (FDM, JN) in order to identify stakeholder roles and the tensions explaining the know-do gap. Secondly, these codes were categorised and coded (JN, FDM) using a coding scheme linked to corresponding slots in the innovation system matrix as outlined in Fig. 1: condition categories (infrastructural, institutional, interactional, capability conditions) and actor-role categories (producers, intermediaries, users, preconditional). This resulted in a categorisation of the data coded during step 1 and more insight into underlying mechanisms. For example, text about reasons mentioned by researchers for not actively sharing knowledge and text originating from a municipal health service interviewee about who he/she thought should coordinate cooperation was coded as ‘institutional’ and ‘intermediary’, as it provided information on the intermediary role. These kinds of text particles formed the tension ‘different perceptions exist about the knowledge broker role’. The actors involved in the tension and the condition category to which it related determined the place of the tension within the matrix. Different types of system conditions were cross-tabulated against the relevant actor categories in order to provide a structured insight into the functioning of the system, as well as to obtain an overview of it and underlying mechanisms shared across actor groups. This completed matrix (see Fig. 2) provides an overview of the Dutch public health innovation system and its key tensions.

During the analytical phase, all authors of this paper had frequent contact and discussed and assessed the outcomes of the analysis. Interviewees all received the final report of this study and were invited to a workshop conference in which the results were presented and discussed.

### Ethical considerations

According to Dutch law, this study did not require formal ethics committee approval, but special attention was paid to informing respondents and protecting their privacy. All participants entered into the research with voluntary consent. They were provided with information about the purpose and contents of the study. Moreover, participants were able to withdraw from the study at any time for any reason. The collected data were treated confidentially and anonymously.

## Results

Figure 2 presents the innovation system matrix (Fig. 1) including the identified tensions explaining the know-do gap and the knowledge broker role. We firstly elaborate on how actor roles (user, producer, intermediary, preconditional) are executed by actors within the Dutch public health innovation system (presented in the top row of the matrix in Fig. 2). Subsequently, the identified tensions and underlying mechanisms are described in more detail, also indicating under which condition (infrastructural, institutional, interactional, and capabilities) the tensions can be placed.

### Actor roles: who does what in the Dutch public health innovation system?

The roles of knowledge producers, knowledge users, intermediaries, and precondition establishers all appeared to exist within the public health innovation system, although it appeared that actors rarely had one single role. According to the interviewees, knowledge was produced mainly by researchers, research and training institutes, and to a lesser extent by consultancy agencies. It appeared, however, that these actors produced different types of knowledge. Researchers and training institutes stated that they mainly produce scientific knowledge (e.g., peer-reviewed papers, reports), and research and training institutes and consultancy agencies asserted that they translated scientific knowledge into easily accessible information (e.g., reports, factsheets, toolkits). Furthermore, from interviewees active at local level it became clear that actors such as employees of municipal health services also produced knowledge. In most cases, this was experience- or practice-based knowledge obtained from their contacts in the field, for example from local policymakers or from the target population. This knowledge was used to adapt their activities (such as interventions) to the local situation. These interviewees, however, did not literally indicate this as producing knowledge.

Research and training institutes and consultancy agencies could, however, also be classified as intermediaries, as knowledge translation is a way of bringing together different types of knowledge. From the interviews it can be concluded that the user role is mostly undertaken by local actors such as municipal health services, municipalities, and citizens, but also by ministries as they use knowledge for policy development. However, as already stated, these local actors combine knowledge products such as reports or toolkits with knowledge from their own experiences and practical circumstances; this makes them knowledge producers also. Preconditions (e.g., determining policy foci, research priorities, and financial systems) for the functioning of the Dutch public health innovation system are established by ministries (mainly the Ministry of Public Health, Welfare, and Sports) and (governmental) funding agencies, and to a lesser extent by research institutes. Policymakers define policy foci mainly on the basis of the current political landscape. This partly guides funding agencies’ priorities in formulating research calls and defining financial systems. Research institutes and universities slightly influence research priorities through the research proposals they write in order to gain funding.

### Key tensions and underlying mechanisms explaining the know-do gap in system condition categories

Seven tensions underlying the know-do gap were identified: research priorities determined by powerful players; no consensus about quality assessment; different perceptions about the knowledge broker role; funding-induced competition engendering fragmentation; thematic funding engendering fragmentation; predominance of passive knowledge sharing; lack of capacity among users to use and influence research.

The tensions relate to infrastructural, institutional, interactional, and capability conditions (see Fig. 2). The circles in Fig. 2 indicate system condition areas in which tensions were observed and the domain of actor roles that cause and/or potentially resolve these tensions (not necessarily reflecting the opinions of the actors involved or affected). We now explain these in more detail.

#### Infrastructural conditions

##### Research priorities determined by powerful players

Interviewees perceived that research priorities are determined by powerful players (such as the Ministry of Health, the Health Council, and research organisations), in isolation from intended users. Interviewees indicated that local policymakers, health professionals, and citizens, or the intended knowledge users, have little influence on the direction and design of the public health and health promotion research agenda.

A majority of interviewees considered that priority setting occurred in relatively closed circles of a small selection of researchers and policymakers with a shared value frame, excluding certain research directions.

The following quote by a municipal health service interviewee illustrates this:

*Why are research results not being used? You could also say to researchers that the research they do is useless if it is not being used. I think our government mainly takes a science perspective and has little consideration for practice.*

The following quotes from an actor at national level and a researcher, respectively, describe how priorities may be determined, indicating that intended knowledge users are not involved in the formulation of policy priorities:

*Sometimes there is just a need for research programmes on a specific topic. These programmes are based on policy made by the minister. You do not always know what it will yield and what the societal impact will be.*

*The real priorities for prevention are largely determined by the Ministry of Health. So according to the policy document on prevention, the focus is on education, health education, and that’s defined by the ministry.*

In terms of the innovation system matrix categories, the contention that research priorities are determined by powerful players (actors in the preconditional domain) is an example of how the knowledge infrastructure is organised, and this can therefore be classified as an infrastructural condition.

##### No consensus about criteria for knowledge quality

Interviewees named various sources of knowledge (research-based, practice-based, experience-based) that they used in their daily work. However, interviewees also felt that there was no consensus on how to weigh the quality of these different types of knowledge: what knowledge should be applied in which situation, and how to integrate the more implicit types of knowledge. The use of various sources of knowledge is illustrated by the following quotes by an interviewee from a municipal health service, which indicate that an actor at national level (health inspectorate) prefers research-based knowledge (first quote), whereas at regional level actors use experience-based knowledge (based on opinions and experience of different actors) (second quote):

*There was this head inspector [health inspectorate] who gave a keynote speech at this conference. He said that our work should be more evidence based.*

*We used to organise a meeting with for example eight organisations from different areas in The Netherlands. … If we had profound disagreements about issues, we would look for literature, but if there were no profound disagreements, we would have like a ‘consensus guideline’, which we would disseminate. That’s much quicker, less evidence based of course, but much quicker.*

There was also no consensus about the existing quality system for effective interventions because interventions assessed as effective were often perceived as not matching local circumstances. From the interviews it became clear that quality systems (e.g., systems for assessing health promotion interventions) were developed in order to set standards for knowledge quality assessment. However, local interviewees (from municipal health services, outside the academic world) did not agree with the-academically-set criteria for these quality systems, because they did not match local (implementation) circumstances.

Another example of distinct opinions about knowledge quality emerged from interviews with researchers, who indicated that, in the academic world, publishing in scientific journals is a top priority. Fundamental research has precedence over practice-based research, and adherence to randomised controlled trials is considered to be the gold standard for creating explicit, systematic, and replicable knowledge. This undervalues practice-based research such as participatory research in real-life settings and experiential knowledge, and does not take into account whether research methods fit the practical context. This becomes clear from the following quotation:

*We [researcher] thought that a randomised controlled trial was needed, but they [health professional] thought that this would harm practice.*

This quote implies that, if quality systems for interventions require a randomised controlled trial as proof of the intervention’s quality, many practice-fit interventions will not be qualified by the system as good.

The following is another example of mismatching ideas about effective interventions. Regional actors indicated that local public health and health promotion professionals prefer knowledge products based on best principles, rather than the available knowledge products with predetermined implementation plans that are prescriptive in nature, often offered by research and training institutes. The following quote from a municipal health service actor conveys this:

*Sometimes it is so specific that you wish the products to be described more broadly so that you do not need a separate script for each target group. … You can also say: the influential mechanism or the most important ingredients … are this and that. Then we do not need separate scripts for migrants, women, children, etc.*

In terms of the innovation system matrix categories, the contention that no consensus exists about criteria for knowledge quality is an infrastructural problem as it gives information about how the knowledge infrastructure is organised. However, it also reflects institutions (hard and soft) relating to how to assess knowledge, which is all about rules and norms. This point involves all actors in the system.

#### Institutional conditions

##### Different perceptions of the knowledge broker role

The interviewees had different opinions about which roles should be played by researchers and training institutes, local actors (such as municipalities and municipal health services), and governmental or consultancy agencies.

Research and training institutes themselves felt little responsibility for integrating implicit and/or practice-based knowledge in their work; and integrating and co-creating activities (e.g., efforts like connecting stakeholders, advocacy, and sharing successes) were not perceived as a formal part of their job description. Local actors, on the other hand, generally thought that researchers should put more effort into active knowledge exchange, thereby combining knowledge from practice (obtained from local actors) with scientific knowledge. Researchers who did make attempts to stimulate active knowledge exchange felt they were not rewarded for this and said that they had not enough time to combine producing knowledge with active knowledge exchange. Additionally, interviewees mentioned a disincentive to engage in multidisciplinary and participatory research (in which research-, practice-, and experience-based knowledge is combined), because this type of research is harder to publish. This problem is described in the following quotation:

*If I [researcher] was only busy writing scientific articles, then my ratings as a scientist would be much higher in terms of the H-index, citations, numbers, etc. Those are the criteria on which we are judged in terms of funding, but that [writing articles] is not the only reason for us.*

Local actors such as municipalities and municipal health services should be, according to actors establishing preconditions, more active in demanding the research- or practice-based knowledge they need. Local actors themselves, however, indicated that a proactive search for knowledge is not obvious in the daily practice of health professionals and local policymakers, and that this inhibits active knowledge exchange. They preferred a situation in which others (like governmental or consultancy agencies) help them to obtain knowledge (often research-based knowledge, as practice-based knowledge is obtained more automatically), for instance by facilitating active interaction with knowledge producers such as researchers. This is expressed in the following quote:

*There is a need for an organiser or a coordinator with dedicated time and means and who brings together different parties.*

Some researchers were more sceptical about this coordinator role for governmental or consultancy agencies, as the neutrality of these agencies was questioned. This connects to the extent to which intermediaries act as messengers following a certain research or policy focus, and thus have an economic stake in selling certain knowledge.

Interviewees representing governmental or consultancy agencies did not perceive themselves as brokers who bring together different parties; rather, they perceived their task as translating research-based knowledge into easily accessible formats or scientific knowledge into ready-made interventions (bringing together different kinds of knowledge, instead of connecting stakeholders).

Hence, actively sharing, integrating, and using different types of knowledge, although stated to be desirable, is lacking at different sites of the public health innovation system. The following quote from a governmental institute illustrates the desirability of active sharing:

*That’s a responsibility of us all. All players in the field play an important role, both national and local players. They all have to share their experiences.*

In terms of the innovation system matrix categories, the contention that different perceptions exist about the knowledge broker role can be classified under hard and soft institutions, as the different actors adhere to several conflicting rules and regulations (hard) and norms and values (soft) about who should do what. Therefore, it is placed in the middle of the matrix, covering all roles.

##### Funding-induced competition engenders fragmentation

Interviewees mentioned that the fact that competition for research funding in The Netherlands is tough and induced by funding structures inhibits open sharing of information for strategic reasons. This is strikingly conveyed by the following quote from a research and training institute interviewee:

*If you are dependent on funding, you want to protect your expertise. If you work together, you can lose things.*

This view seems to exist only at national level and among consultancy agencies, research and training institutes, and universities (of applied sciences), who mentioned this issue. Local actor interviewees did not mention it.

In terms of the innovation system matrix categories, the contention that funding-induced competition engenders fragmentation reflects both hard institutions (funding structures) inducing competition and soft institutions: the fear of losing valuable knowledge to others. Furthermore, it may also be categorised in strong network conditions as these hard and soft institutions mean that actors are not stimulated to share their knowledge with others outside their own network.

##### Thematic funding engenders fragmentation

From the interviews with the research and training institute representatives it became clear that the way funding is theme- and target group-oriented leads to a situation in which each research and training institute produces its own, theme-related and/or target group-specific (research-based) knowledge (e.g., especially on smoking or migrants). This creates a complex and fragmented knowledge landscape in which it is hard for knowledge users to find the information they need. An interviewee from a municipal health service articulates this:

*It’s a matter of interpretation [of knowledge products such as interventions]. And compare it with what you’re already doing, this has to be accompanied with tailored advice.*

This theme-related and/or target group-specific funding also leaves no room for overarching themes such as advocacy and participation that play a role in several local public health programmes regardless of the health theme. For example, prevention of alcohol abuse or promotion of healthy eating requires similar actors to become involved. Several interviewees stated that they would prefer a general public health infrastructure over a new network for each public health issue, as shown by the following quote from a consultancy agency:

*Personally I do not think it matters what theme you work on. All partners deal with the same issues, sometimes you need to get other partners involved. So certain structures could overlap, the focus is only on another theme.*

Furthermore, the interviewees from municipal health services and from governmental research institutes both mentioned that there is often no congruence in planning horizons and working routines. In particular, the fact that local or national governments often change their policies as a result of elections hampers continuity of health promotion activities, as changes in policy often involve changes in budgets and financial allocations. This indicates that thematic funding induces fragmentation at both national and local level. Additionally, from the interviews it becomes clear that there are many ad-hoc initiatives to create network structures, thereby inducing network fragmentation.

In terms of the innovation system matrix categories, the contention that thematic funding engenders fragmentation reflects the fact that hard institutions such as funding schemes influence knowledge exchange. The tension involves mainly preconditional actors and affects mainly knowledge users.

#### Interactional conditions

##### Predominance of passive knowledge sharing

Different interviewees indicated that, as a result of the lack of interaction between knowledge users and knowledge producers, there appears to be an inadequate match between the supply of, and demand for, knowledge products (often research-based): passive knowledge transfer in the form of reports, databases, and websites is the most common form of implementation. Additionally, more implicit types of knowledge, such as experience-based knowledge, are often not made explicit. An example of this inadequate match and the need for interaction is given in the following quote from a municipal health service interviewee:

*You can make a digital source but that is often not used. When you sit around the table with people who have experience with a certain method or approach, you can ask detailed questions and make further appointments to discuss the details.*

The next quote, from a research and training institute interviewee, also indicates the need for more interaction, especially between the local and national level, and suggests that financial support is needed to create more interaction and active exchange of knowledge:

*We need financial support to facilitate linkages between national players and local municipalities. If we leave this to the market, we will lose it. That will widen the gap between the different levels.*

In terms of the innovation system matrix categories, the contention about the predominance of passive knowledge sharing reflects weak network conditions because knowledge producers do not have intense interaction with users, and this leads to inadequate knowledge exchange.

#### Capabilities conditions

##### Lack of capacity among users to use and influence research

Related to the lack of a quality system, agreed upon by all, is the fact mentioned by interviewees that local health professionals, such as employees of municipal health services, find it difficult to choose the best knowledge, as they often lack the time and the specialised and qualified human capacity to engage in knowledge search and use. The following quotation from a local actor illustrates this:

*People do see the need and are willing, but it [implementing externally obtained knowledge] is another additional task. Everyone is enthusiastic at the start, but at a certain point it’s just a lack of human resources. At national level, this is often underestimated. It is important, isn’t it? Of course it’s important, but many other issues are important as well.*

Moreover, health professionals who wish to engage in research need to meet complicated requirements in order to become involved in a research priority-setting process or to obtain funding. Local policymakers, professionals, and citizens often do not have sufficient competencies or time to write funding proposals, as illustrated by the following quote from a municipal health service interviewee:

*If you wish for something in practice, you nearly always need a research institute to formulate your research question in a way that you are able to obtain funding. That is what I find difficult …, whereas I notice at the same time that they [the funding agency] try to finance the good products and to set quality criteria and that it is important for practice that this happens well.*

Researchers on the other hand are not trained in active knowledge exchange and lack experience in participatory approaches or managing stakeholder interaction. And, as already mentioned, researchers and training institutes are not rewarded for - and therefore do not have time/capacity for - integrating implicit and/or practice-based knowledge in their work.

In terms of the innovation system matrix categories, the lack of capacity among users to use and influence research reflects the lack of time and manpower (to assess, collect, and use scientific knowledge and to influence the research agenda). It can therefore be classified under capabilities conditions.

## Discussion

As can be concluded from Fig. 2, the most prominent tensions influencing the know-do gap and the functioning of the public health innovation system can be summarised as: research priorities determined by powerful players, no consensus about criteria for knowledge quality, different perceptions about the knowledge broker role, funding-induced competition engenders fragmentation, thematic funding engenders fragmentation, predominance of passive knowledge sharing, and lack of capacity among users to use and influence research.

### The strong influence of institutions on collaboration for innovation

Figure 2 also shows that a large amount of tension is related to institutional conditions and includes almost all players in the field. This affirms the crucial importance of institutions as rules of the game within knowledge exchange processes. This encompasses not only those institutions that affect knowledge production and use such as publication incentives or planning horizons; those that affect the opportunity to engage in cooperative work in a broader sense should also be taken into account. Examples originating from this study are: funding schemes and access to research and innovation agenda-setting procedures, perceptions of what is good knowledge (quality criteria), perceptions on what actors are relevant, and the organisational space provided in terms of time input and capacity building. This importance and broadness of institutions is also indicated by other literature in which institutions are considered as enabling and constraining factors-set by the broader prevailing social, economic, and political context-that determine how people select information, how they interact, and do or do not cooperate [5, 7, 36]. Institutions are described as norms, values, rewards, and incentive structures that may determine the organisation of research financing, the way research priorities are set, and whether knowledge is perceived as ‘good’ knowledge or not (scientific knowledge is often perceived as better knowledge) [5, 7, 36].

### Innovation requires broad linkage building at several interfaces: a need to support networking by systemic intermediaries

From Fig. 2 it can be concluded that passive communication (passive knowledge exchange) to make research results available through written material, databases, and protocols (transfer of research results) receives far more emphasis in The Netherlands than active exchange through interaction efforts between the public, policymakers, intermediaries, and research and training institutes. Our results show a high level of fragmentation within the public health sector. The various actors produce or use knowledge in isolation from other actors (for example only regarding knowledge on their ‘own’ theme or only involving one type [scientific/practical/experiential] of knowledge). This lack of interaction may be explained by an overemphasis on the differences (distinct rationales, incentives, institutions) between different actor domains, a priori assuming that interaction is difficult or impossible, and implying that the distinction between domains is not changeable [37]. Fig. 2 also shows that many tensions involve the intermediary role: the intermediary role is somewhat underrepresented or has limited role interpretation, indicating that no actor feels responsible for facilitating active knowledge exchange or for reducing distinctions between domains. This is in line with the large body of scientific literature on barriers to, and facilitators of, effective knowledge exchange, with a strong bias towards research as the only legitimate source of knowledge, a linear view on knowledge exchange as diffusion with a limited focus on bilateral or multilateral interfaces [38]. However, lately these views have been changing: there is a recognition of the multi-actor, systemic nature of innovation processes and the fact that there are multiple producers of useful knowledge beyond scientific knowledge [9, 18, 39–41]. This is referred to as knowledge co-creation, a situation in which actors cooperate in order to combine different types of knowledge (cooperation results in jointly created knowledge) [12–16, 42, 43]. The identified underrepresentation or limited interpretation of the intermediary role may thus indicate a lack of facilitation of co-creation. Bodies of knowledge that may be combined in knowledge co-creation relate to, for example, traditional types of knowledge (such as scientific knowledge, policy) and practice-based knowledge on how research-based knowledge functions in specific local contexts [41, 43].

Moreover, this study supports the notion from innovation systems thinking [16, 19] that innovation is much more than just implementation of research results, but entails well-functioning multi-actor networks that differ in size and complexity depending on the issue at stake, as reflected by the interactional conditions of the innovation system framework used for this study [28]. This calls for acceptance that effective knowledge exchange is essentially about stimulating the formation and functioning of such networks. Integrated efforts to enable knowledge co-creation, coupling research-based, experience-based, and practice-based knowledge, can be created in so-called knowledge platforms [39]. In that case, research is one among many different stakeholders, equally contributing with knowledge, be it scientific or experiential. The lack or insufficiency of such networks may explain some of the findings of this study: why there is a lack of consensus about what is ‘good’ knowledge and about the knowledge broker role (within such networks all types of knowledge would be made relevant and roles could be discussed and agreed upon), why research priorities are determined by powerful players, and why users are not involved in agenda setting (within such networks, priorities would not be set in isolation from other players). Such multi-actor networks (knowledge platforms) self-organise because many actors react to one another’s actions in unpredictable ways [8, 15, 19, 21, 44]. Therefore, knowledge can never be offered in a fixed format [38] but has inherently dynamic properties since it is shaped in interactions [37, 45]. In order to enable such interactions, more attention should be paid to shaping such multi-actor networks, which may be small at the local level, for example by involving consultants, practitioners, policymakers, and citizens, or large when they deal with overarching issues relevant for whole sectors [26, 46]. Such multi-actor networks also imply more diversity in the nature of roles as all actors may assume different roles (producer, user, intermediary, preconditional) at the same time or may assume different roles on different occasions. This requires the knowledge broker role to be aimed much more at stimulating productive interactions instead of ‘pushing’ research knowledge, which is also still prevalent in the Dutch public health system as our study found, and the knowledge broker to become what has been called a systemic intermediary or innovation broker [47–49]. In The Netherlands, examples of attempts to create such multi-actor networks (knowledge platforms) include academic collaborative centres, which are structural collaborations between universities and municipal health services [5, 35, 37] with dedicated systemic intermediation to optimise interactions.

### The need for a shift from being mere research funders to being innovation funders

Whereas in this study we have focused mainly on the knowledge component of innovation systems, recent innovation systems research has highlighted the fact that innovation systems - beyond knowledge creation, exchange, and use – need to fulfil several other functions that are essential for innovation. These functions include fostering entrepreneurial drive and activity, vision development, financial resource mobilisation, market formation, building legitimacy for change, and overcoming resistance to change by means of advocacy and lobbying [14, 19, 41, 50]. Hence, several additional activities beyond production and exchange of knowledge play a key role, such as policy and legislation formulation, physical infrastructure building or adaptation, creating or adapting innovation funding arrangements, and making use of market developments. Funding agencies should more actively influence these activities if they want to support research use [2, 51]. If funding agencies want to enhance the applicability of research and ensure that overall innovation takes place, they should go beyond being research funders and become innovation funders (see [36, 42, 52]). This implies that they should act as connectors and matchmakers in fragmented knowledge infrastructures overseeing other intermediaries and have a presence at many innovation system levels. They should also address several other gaps that are unrelated to the use of research knowledge, such as overcoming the identified tensions within weak and strong network conditions, and mediate to resolve tensions within hard and soft institutional conditions. So beyond being funders financing knowledge creation and exchange, they have a broader task as innovation brokers [45, 53] facilitating a multilateral dialogue and fostering the emplacement of preconditions for innovation and the mitigation of system failures.

### Strengths and limitations

A strength of this study is that, to our knowledge, it is the first to apply an innovation systems approach to analyse knowledge production and use, as well as the contextual factors that influence whether and how effective learning (use of knowledge) takes place for innovation. Previous work has not been as elaborate as this study. The systemic and multi-actor nature of innovation processes has been translated into comprehensive perspectives.

However, the innovation systems view was applied in response to a feeling of suboptimal knowledge use in a sectorial system of innovation (public health) and focused mainly on the function of knowledge production and exchange in innovation systems, whereas recent literature has recognised that innovation systems need also to fulfil other functions apart from stimulating knowledge production and exchange, such as the creation of visions of how the system should progress and mobilising resources to foster innovation [19, 30]. The other functions of innovation systems and also the different organisational levels present within organisations, such as strategic and operational levels [5], fell outside of the scope of this study. However, (a lack of) some of the other functions innovation systems should fulfil, such as resource mobilisation and vision formulation (e.g., via agenda setting), did emerge in the analysis. Hence, the study can be seen as a starting point for more in-depth studies on country-level conditions for innovation in public health.

Using the innovation system matrix as a framework for analysing data can also be considered a strength of this study, as the matrix appeared to be very useful for structuring and overviewing the data. All tensions could be placed under at least one of the four condition categories, and no additional categories were needed. However, the matrix appeared to be less useful in mapping possible interrelatedness between the tensions or the actors, as its layout sometimes forced artificial separations. A model in which actors were not fixed to the upper row of the matrix but could be for example flexibly connected to condition categories to which they contribute would better enable envisioning possible actor interrelatedness. It might also be helpful to add or remove actor categories, depending on the subject of study and the actors involved. From our experiences with this study, the condition categories do not need any alteration, as they enabled characterisation of all the identified tensions.

Furthermore, the fact that a limited number of representatives were interviewed may have influenced the external validity of the findings. However, validity-improving actions were taken: a large variety of representatives was included, and during the interviews data saturation occurred. Moreover, interviewees all received the final report of this study and were invited to a workshop conference in which the results were presented and discussed. That this did not result in new information indicates a high validity. Moreover, feedback received on presentation of the study results at research-or practice-based conferences yielded confirmation of the findings.

It should also be noted that the use of semi-structured interviews may have resulted in data that focused on research-based knowledge. The explicit character of this type of knowledge may have caused interviewees to talk more easily about research-based knowledge, rather than experience- or practice-based knowledge. A more observational method may have been better suited for this research. On the other hand, observational methods (such as observing how someone looks for information) would have influenced the way in which participants in the study acted; it may also be practically unfeasible as it would require in-depth ethnographic work. Additionally, the fact that our results were confirmed by stakeholders from the Dutch public health sector during both research-focused and practice-focused conferences suggests that the methods used were appropriate to capture the knowledge exchange dynamics and the conditions that enable or disable it.

Finally, as mentioned in the results section of this paper, the data for this study were collected in 2009. Since then, some changes that may have taken place within the Dutch public health sector may influence the practical relevance of this study. The Dutch National Programme on Prevention (2014–2018) proves, however, that the findings of this study are still relevant. Within this programme, The Netherlands Organisation for Health Research and Development refers to this study and emphasises the importance of the interaction of different actors and the use of different types of knowledge for effective public health action [54]. The outcomes of this programme will indicate whether this approach has been effective in better closing the know-do gap and improving overall interaction in the Dutch public health innovation system.

## Conclusions

The aim of this study was to identify key tensions and underlying mechanisms explaining the know-do gap in the Dutch public health sector and, by using the innovation system framework, to investigate how they relate to system conditions and actor roles. The perceived know-do gap in The Netherlands consists of a complex mixture of causes and effects, and involves a large variety of actors.

An important first step towards improving public health innovation and effective knowledge exchange, thereby contributing to bridging the know-gap, is to assign a more central role to users, acquire a better understanding of the user context, provide better support structures for integration and collaboration, and create contextual conditions for knowledge to become more effective. Opportunities for linking research to action can be found in improving users’ capabilities to absorb several kinds of knowledge from several sources in order to resolve specific issues at hand (i.e., form the right knowledge networks) and in improving producers’ skills to connect and interact with users and intermediaries who facilitate interaction. Funding agencies (preconditional), research and training institutes, and consultancy agencies (intermediaries) could have a role in creating better linkages and actively managing interaction for knowledge co-creation.

We directed our analytical focus at the functioning of the Dutch public health sector, as this case may be exemplary for knowledge exchange within other (public health) sectors.

Beyond the context of the Dutch public health system, the generic message of this article relates to its application of a systemic perspective on knowledge production, exchange, and use. Rather than just speaking about linking research to action and bridging the know-do-gap, an innovation systems view, in which research is one amongst many stakeholders, implies speaking in terms of linking all kinds of actors in order to enable co-creation of knowledge and removing institutional barriers to innovation. The systemic view results in a more comprehensive picture of the barriers to, and facilitating factors for, innovation and change.
